# Deep Learning Application for Analyzing of Constituents and Their Correlations in the Interpretations of Medical Images

**DOI:** 10.3390/diagnostics11081373

**Published:** 2021-07-30

**Authors:** Tudor Florin Ursuleanu, Andreea Roxana Luca, Liliana Gheorghe, Roxana Grigorovici, Stefan Iancu, Maria Hlusneac, Cristina Preda, Alexandru Grigorovici

**Affiliations:** 1 Faculty of General Medicine, “Grigore T. Popa” University of Medicine and Pharmacy, 700115 Iasi, Romania; tudorursuleanu@yahoo.com (T.F.U.); roxanagrigorovici@yahoo.com (R.G.); istefan81@gmail.com (S.I.); mariahlusneac0@gmail.com (M.H.); cpreda1@yahoo.com (C.P.); alexandrugrigorovici@yahoo.com (A.G.); 2 Department of Surgery VI, “Sf. Spiridon” Hospital, 700111 Iasi, Romania; 3 Department of Surgery I, Regional Institute of Oncology, 700483 Iasi, Romania; 4 Department Obstetrics and Gynecology, Integrated Ambulatory of Hospital “Sf. Spiridon”, 700106 Iasi, Romania; 5 Department of Radiology, “Sf. Spiridon” Hospital, 700111 Iasi, Romania; 6 Department of Endocrinology, “Sf. Spiridon” Hospital, 700111 Iasi, Romania

**Keywords:** medical image analysis, types of data and datasets, methods of incorporating knowledge, deep learning models, applications in medicine

## Abstract

The need for time and attention, given by the doctor to the patient, due to the increased volume of medical data to be interpreted and filtered for diagnostic and therapeutic purposes has encouraged the development of the option to support, constructively and effectively, deep learning models. Deep learning (DL) has experienced an exponential development in recent years, with a major impact on interpretations of the medical image. This has influenced the development, diversification and increase of the quality of scientific data, the development of knowledge construction methods and the improvement of DL models used in medical applications. All research papers focus on description, highlighting, classification of one of the constituent elements of deep learning models (DL), used in the interpretation of medical images and do not provide a unified picture of the importance and impact of each constituent in the performance of DL models. The novelty in our paper consists primarily in the unitary approach, of the constituent elements of DL models, namely, data, tools used by DL architectures or specifically constructed DL architecture combinations and highlighting their “key” features, for completion of tasks in current applications in the interpretation of medical images. The use of “key” characteristics specific to each constituent of DL models and the correct determination of their correlations, may be the subject of future research, with the aim of increasing the performance of DL models in the interpretation of medical images.

## 1. Introduction

The performance of deep learning architectures (DL) has a continuously improved by increasing the number and quality, respectively diversification data resources similar to medical data, developing specific methods of integrating data into DL models according to the objectives for which they were built and perfecting the construction of DL models used in medical applications.

Deep learning (DL) has experienced an exponential development of medicine, but applications in interpretations of medical imaging are in continuous development. DL has managed to achieve performance in diagnosis, classification, detection, segmentation, reconstruction of medical images [1] but also in achieving the correlation between image diagnosis and patient survival, predicting new directions of development [2]. 

The novelty in our paper consists in the unitary approach, of the constituent elements of DL models, namely, data, tools used by DL architectures or specifically constructed DL architecture combinations and highlighting their “key” features, for completion of tasks in current applications in the interpretation of medical images. 

In this article we present in primarily, a unitary, complete, up-to-date analysis of scientific data, methods of knowledge incorporation, a classification and description of DL models according to the structure and objectives for which they were designed and presentation of medical applications according to these tasks. Secondly, it describes the specific correlations between data, data integration methods, deep learning models used in the interpretation of diagnostic medical images and their applications in medicine. Finally presents problems and future challenges.

The structure is composed of Section 2 describes types of images, medical data used by deep learning architectures, Section 3 describes DL models according to the objectives for which they were created, medical application, associating the types of data, Section 4 methods of incorporating images, information and medical data, in addition to the objective of DL. Section 5 contributions of the methods of incorporating images, information and medical data in medical applications, Section 6 research issues and future challenges.

**Figure 1:**
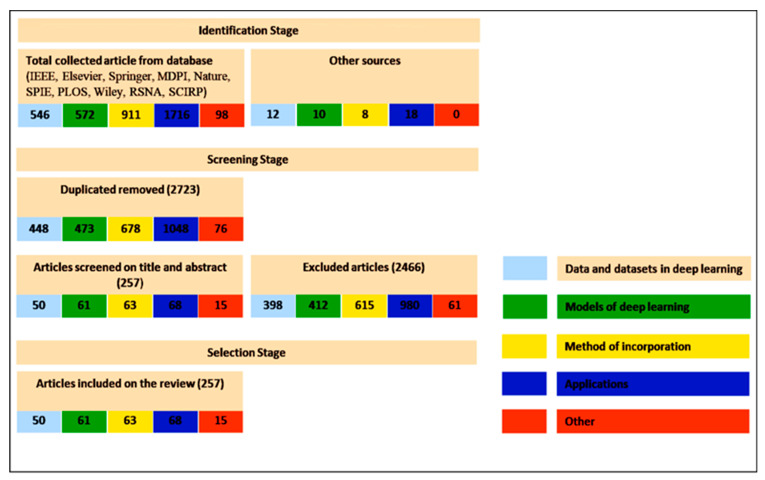
Search Framework.

Methodology:

We have identified and selected significant research papers published in 2009–2020, mainly from 2016 and 2020, with some papers from 2021. We focus on papers from the most reputable publishers, such as IEEE, Elsevier, Springer, MDPI, Nature, SPIE, PLOS, Wiley, RSNA, SCIRP. Some works have been selected from arXiv. I have reviewed more than 273 papers on different DL topics. There are 17 works from 2021, 56 works from 2020, 56 works from 2019, 38 works from 2018, 58 works from 2017, 25 works from 2016 and 10 work from 2015. This indicates that this review focus on the latest publications in the field of DL. The selected papers have been analyzed and reviewed for: descriptions types of images, medical data used by deep learning architectures (Section 2), descriptions DL models according to the objectives for which they were created, medical application, associating the types of data (Section 3), methods of incorporating images, information and medical data, in addition to the objective of DL (Section 4), contributions of the methods of incorporating images, information and medical data in medical applications (Section 5), research issues and future challenges (Section 6). Most keywords used for search criteria for this review work are (Deep Learning and Data types), (Deep Learning and Data Sets), (Deep Learning and Methods of Incorporation of Medical Knowledge and Data), (Deep Learning Models and Models), (Deep Learning and Architectures), ((Deep Learning) and (Medical Image Analysis) and (Detection/Classification/Segmentation/Localization/Reconstruction/Recovery)), (Deep Learning and Detection/Classification/Segmentation/Localization/Reconstruction), (Deep Learning and Images and Applications in Medicine), (Deep Learning and Interpretation Medical Images). Figure 1 shows our search structure of the survey paper.

## 2. Scientific Data and Dataset

### 2.1. Types of Images and Datasets in the Medical Domain

Medical data, types of images, images from time series, audio-video data represent unstructured information have a need for labeling because they make the process of data extraction difficult because they suffer high levels of noise and variability, and classical deep learning architectures achieve low performance in interpretations of medical images.

The interpretation of medical images in diagnostic radiology through the use of deep learning architectures has applications in cancer diagnosis, with satisfactory results in the diagnostic detection of breast cancer, lung cancer, glaucoma and skin cancer.

**Figure 2:**
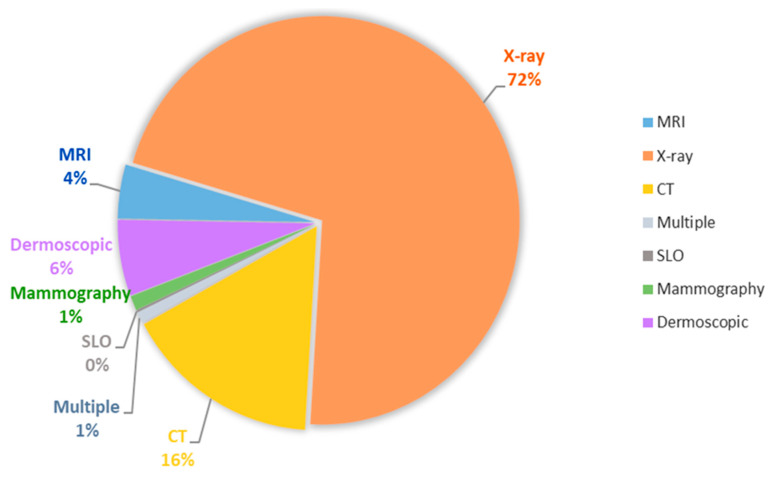
Imaging and exploratory investigations in the process of image interpretation.

CT, PET-CT, MRI, X-rays, Ultrasound, Diagnostic Biopsy, Mammography and Spectrography are the most used imaging and exploratory investigations in the process of image interpretation, in the objective of extracting characteristics, reducing or enlarging the size, in the group, segmentation and classification of images and by using integration methods contribute to the performance of deep learning models, see Figure 2 [3].

Acronyms: MRI Magnetic Resonance Images, CT Computed Tomography, SLO Scanning Laser Ophthalmoscopy images, X-ray on weakly-supervised classification and localization of common thorax diseases.

**Figure 3:**
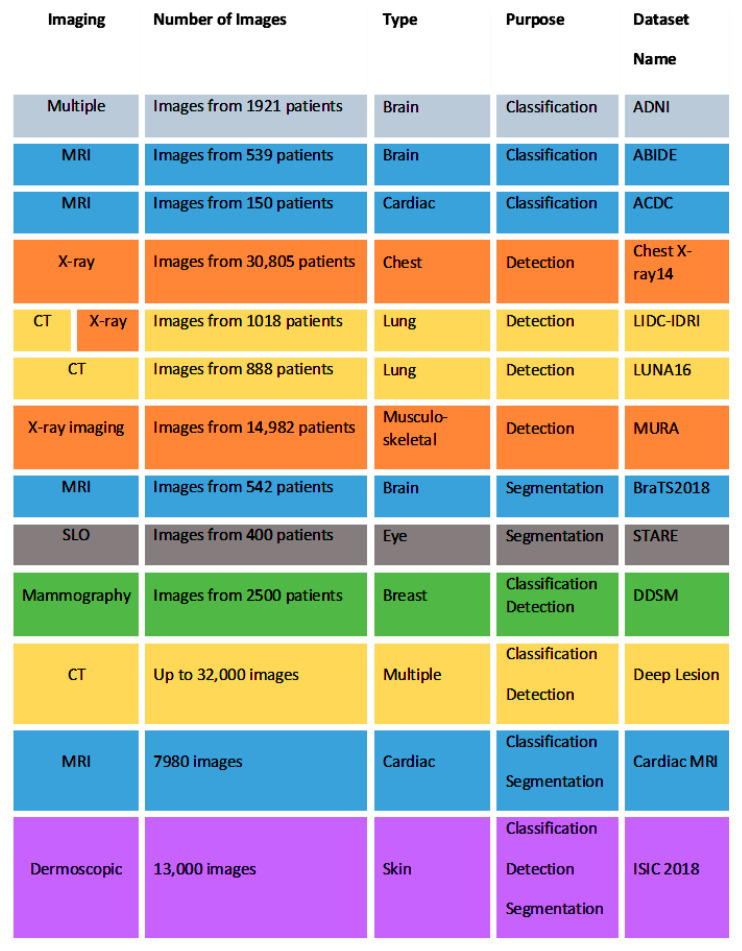
Types of images datasets in the medical domain.

Larger datasets, compared to the small size of many medical datasets, result in better deep learning models [4]. The large and well-annotated data sets are: ImageNet, COCO 2, (open source) medical data sets, see Figure 3.

Acronyms: MRI Magnetic Resonance Images, CT Computed Tomography, SLO Scanning Laser Ophthalmoscopy images, The Alzheimer’s disease neuroimaging initiative (ADNI), Automated cardiac diagnosis challenge (ACDC), The autism brain imaging data exchange (ABIDE), Hospital-scale chest x-ray database and benchmarks on weakly-supervised classification and localization of common thorax diseases (Chestx-ray14), The lung image database consortium (LIDC) and image database resource initiative (IDRI) (LIDC-IDRI), Algorithms for automatic detection of pulmonary nodules in computed tomography images (LUNA16), Large dataset for abnormality detection in musculoskeletal radiographs (MURA), Machine learning algorithms for brain tumor segmentation, progression assessment, and overall survival prediction in the brats challenge (BraTS2018), Locating blood vessels in retinal images (STARE), Digital database for screening mammography (DDSM), Automated mining of large-scale lesion annotations and universal lesion detection with deep learning (DeepLesion), Cardiac Magnetic Resonance Images (Cardiac MRI), International skin imaging collaboration (ISIC).

The knowledge of experienced clinical-imagists, follow certain characteristics in images, namely, contrast, color, appearance, topology, shape, edges, etc., contributes to the performance of medical image interpretation through the use of deep learning models, namely, anomaly detection by identifying the characteristics in the image; image segmentation; image reconstruction; combining two different images into one [5].

The knowledge of imaging doctors can be classified as follows:

1. Low-level medical data
  - Areas of attention of physicians in medical images [6],
  - Disease characteristics [7],
2. High-level medical data
  - Labels–Diagnostic pattern [8],
3. Diagnostic training model that represents specific data identified by doctors [9].

The type and volume of medical data, the labels, the category of field knowledge and the methods of their integration into the DL architectures implicitly determine their performance in medical applications.

### 2.2. Types of Images and Medical Data Used for Diagnosis–Classification of Diseases in Medical Images

We will further expose, the types of medical images and data used in diagnosis-classification, segmentation, detection, reconstruction, recovery and, respectively, the generation of medical reports.

Natural images–from natural datasets, ImageNet 1 (over 14 million images tagged in 20 k categories) and COCO 2 (with over 200 images annotated in 80 categories).

Medical images-from medical datasets of the same diseases in similar and different ways or from different diseases [10].

High-level medical data (diagnostic pattern), low-level medical data (areas of images, disease characteristics).

Specific data identified by doctors (attention maps, hand-highlighted features) increase the diagnostic performance of deep learning networks (no comparative studies have been conducted).

### 2.3. Types of Images and Medical Data Used for Diagnosis Detection of Lesions and Abnormalities in Medical Images

Large natural images (ImageNet) are incorporated for the detection of characteristics in the medical images. Natural images are used in multiple applications.

Medical images are used in multiple applications. Multi-modal medical images, PET images are incorporated for the detection of lesions in CT scans.

High-level medical data (diagnostic pattern), low-level medical data (areas of images, disease characteristics).

Specific data identified by doctors (attention maps, hand-highlighted features) increase the diagnostic performance of deep learning networks (no comparative studies have been carried out).

### 2.4. Types of Images and Medical Data Used for Diagnosis–Segmentation into Medical Images

Natural Images, ImageNet, PASCAL VOC “static data” set, Sports-1M video datasets [11].

Medical images, (CT, MRI, Angio-CT, butt eye images, annotated retinal images) used in multiple applications.

External medical data and images of other diseases, dataset 3DSeg-8 [12].

High-level and low-level medical data, e.g., anatomical aspects of the image, shape, position, typology of lesions integrated into segmentation tasks, example of the ISBI 2017 dataset used in skin injury segmentation. Many applications use additional data with satisfactory results to improve CT image segmentation tasks in order to improve applications for MRI use [13].

Medical data from doctors, hand-made features, hand-highlighted features, are first processed from the reference images. These features are used in the BRATS2015 dataset in input-level merging image segmentation applications.

### 2.5. Medical Data and Manual Features Used for Image Reconstruction

X-ray projections in CT or spatial frequency information in MRI) [14,15], image reconstruction with optical diffuse tomography (DOT), reconstruction of magnetic resonance imaging by compressed detection (CS-MRI) [16], reconstruction of the image with diffuse optical tomography (DOT) of limited-angle breast cancer and limited sources in a strong scattering environment [17,18], recovery of brain MRI images, target contrast using GAN [19] are methods based on deep learning have been widely applied in this area.

### 2.6. Medical Data and Manual Features Used for Image Recovery

Knowledge from natural images, medical datasets for example, age and sex of patients, characteristics extracted from health areas.

### 2.7. Medical Data Used to Generate Medical Reports

Subtitling medical images, templates from radiologist reports, visual characteristics of medical images, generating reports using the IU-RR dataset.

## 3. DL Models Description and Classification According to the Tasks in Medical Images Analyses

We will describe the deep learning architectures in relation to the purpose and tasks for which they were designed, namely, diagnosis-classification, detection, segmentation, reconstruction.

### 3.1. DL Architectures Designed for Diagnosis–Classification in Medical Images

CNN, AlexNet, GoogLeNet, VGGNet, ResNet, DenseNet are used for diagnosis, classification, diseases.

GoogLeNet, VGGNet, ResNet are used for diagnosis, classification of superficial and deep corneal ulcers with accuracy of over 90%.

DenseNet [20] used for diagnostic classification of lung nodules on X-Rey with accuracy of over 90% Architectures designed to detect objects in natural images used to detect objects in medical images.

### 3.2. DL Architectures Designed for Diagnosis Detection of Lesions, Abnormalities in Medical Images

Two-stage models for injury and organ detection consist of a network of regional proposals (RPN) that involves the locations of candidate objects and a detection network that selects regional proposals are Faster R-CNN [21] and Mask R-CNN [18,22].

Models with a faster and simpler stage, which go over the stage of the proposal of the region and run the detection directly, taking into account the probability that the object will appear at every point in the image such as YOLO (You Only Look Once) [23], SSD (Single Shot MultiBox Detector) [9] and RetinaNet [24].

Combined FCN and GAN architectures, through PET images are generated first from CT scans then synthesized PET images are used in a false positive reduction layer [18,25].

### 3.3. DL Architectures Designed for Diagnosis Segmentation of Medical Images

Three categories can be exemplified: FCN-based models [26]; U-Net-based models [27]; GAN-based models [28].

#### 3.3.1. FCN Achieves Goals of Segmenting the Medical Image with Good Results 

Types of FCN: Cascading FCN [29,30], parallel FCN [31] and recurrent FCN [32] also achieve medical image segmentation goals with good results.

#### 3.3.2. U-Net-Based Models

U-Net [27] and its derivatives segment the medical image with good results. U-Net is based on the FCN structure, consisting of a series of convolutional and devolutionary layers and with short connections between equal resolution layers. U-Net and its variants such as UNet ++ [33] and recurrent U-Net [34] perform well in many medical image segmentation tasks [18,35].

#### 3.3.3. GAN-Based Models

GAN is a type of mixed architecture (supervised and unsupervised) called semi-supervised architecture, an architecture composed of two neural networks, a generator and a discriminator or classifier, which compete with each other in a contradictory formation process [28]. In models, the generator is used to predict the target mask based on encoder-decoder structures (such as FCN or U-Net) [18]. The discriminator serves as a form regulator that helps the generator achieve satisfactory segmentation results [16,33]. GAN has use in the generation of synthetic instances of different classes.

### 3.4. DL Architectures Designed for Diagnosis, Classification, Segmentation, Detection and Reconstruction of Medical Images

Deep auto-encoders (AUD) are included in the type of unsupervised learning that uses unlabeled input data, there is no a priori knowledge, and the results to be obtained from the processing of input data are unknown, and can learn to organize information without providing an error calculation to evaluate the possible solution [36,37]. The main feature of the autoencoder is represented by the input and output layers have the same size, and the output must reproduce the input, while the hidden layers are smaller in size because the input patterns are progressively encoded and decoded throughout the process, and has the ability to extract the fundamental characteristics of the input, being used to reduce the size of the data, but also to reduce noise in input data (such as images). They are often used for data reconstruction (image and signal), denoising or augmentation [37,38].

### 3.5. Medical Applications of DL Models According to the Scope for Which They Were Used, Classification, Segmentation, Detection and Reconstruction of Medical Images

DL architectures, e.g., CNN, U-Net, ResNet, VGGNet, AlexNet, RNN, GAN, DBN, YOLO and respectively, the types of combined architectures VGGNet + CNN, CNN + LSTM, GAN + U-Net, VGGNet + U-Net, RCC + U-Net which have as tasks classification, segmentation, detectionand reconstruction of medical images are the most used and have the best performance and contribution to medical applications (see Table 1) [39].

## 4. DL Model Description and Classification According to Medical Data Types Used, Objectives and Performances in Medical Applications

### 4.1. DL Models According to the Characteristics and Tasks for Which They Were Designed

CNN (convolutional neural network) are popular in areas where the shape of an object is an important feature, such as image analysis [5,39,94,95], particularly in the study of cancers and bodily injuries in the medical sector [96,97] and video analysis [39,98].

CNN contains convolutive layers, grouping layers, dropout layers, and an output layer, hierarchically positioned that each learn stun specific characteristics in the image [99].

CNN in image analysis has low performance when high-resolution datasets are considered [100] and when localization over large patches is required, especially in medical images [101,102].

**Figure 4:**
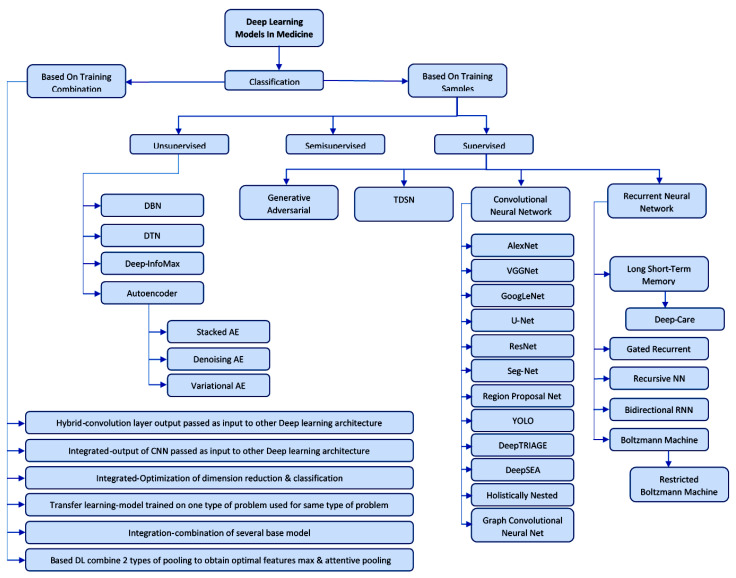
Classification of DL models according to the characteristics and tasks for which they were designed. Acronyms: Deep Network of Beliefs (DBN), Deep Network of Distribution and Target, Deep Info Max (DIM), AutoEnconder (AE), Generative Adversarial Network (GAN), Tensor Deep Stacking Network (TDSN), Convolutional Neural Network (CNN), Visual Geometry Group Network (VGG Net), Deep Layers Network (GoogLeNet), Fully Convolutional Network (U-Net), Residual Neural Network (ResNet), Deep Segmentation-Emendation Network (SegNet), Region Proposal Net (RPN,), You Only Look Once (YOLO), Deep Triage (DT), deep learning–based algorithmic framework (DeepSEA), Holistically-Nested Edge Detection (HED), Graph Convolutional Natural Net (GCNN),Recurent Neuronal Network (RNN), Deep Dynamic Neural Network (Deep Care),Gated Recurrent Network (GRN), Recurrsive RNN(RvNNs), Long Short-Term Memory (LSTM), Bidirectional RNN (BRNN), Restricted Boltzmann Machine (RBM).

We will synthesize in Figure 4 Classification of DL models according to the characteristics and tasks for which they were designed, classification of DL models according to the characteristics and tasks for which they were designed and describe them later [102].

DL architectures classification [103]:

Supervised DL models:

- Recurrent Neural Networks (RNN), Long short-term memory (LSTM), Gated Recurrent Unit (GRU),
- Convolutional Neural Network (CNN)
- Generative Adversarial Network (GAN).

Unsupervised deep learning models:

- Deep Network of Beliefs (DBN),
- Deep Transfer Network (DTN),
- Tensor Deep Stack Networks (TDSN),
- Autoencoders (AE).

CNN’s performance is strongly influenced by the selection of hyper-parameters. Any small changes in hyper-parameters will affect CNN’s overall performance. Therefore, careful selection of parameters is an extremely significant problem that should be taken into account during the development of the optimisation scheme.

Impressive and robust hardware resources, such as GPs, are needed for an effective CNN workout. Moreover, they are also needed to explore the effectiveness of using CNN in intelligent and embedded systems.

Exploitation of depth and various structural adaptations is significantly improved in CNN’s learning capacity. Replacing the traditional layer configuration with blocks leads to significant progress in CNN’s performance, as shown in recent literature. Today, the development of new and efficient block architectures is the main trend in the new research models of CNN architectures. HRNet is just one example that shows that there are always ways to improve the architecture. Cloud-based platforms are expected to play a key role in the future development of DL computing applications [104].

Several deep learning, computer assisted diagnosis (CAD) systems for digital breast tomosynthesis (DBT) are currently available and many new systems will be developed. However, there are still many challenges to overcome. As Wang et al. [105] have recently demonstrated, published models for the full-field digital mammography (FFDM) classification fail when applied to different datasets, even when these data sets include purchases using similar equipment. For FFDMs, deep learning-based detection models have proven to be performing with almost human precision [106]. As more studies and data become available, there is no reason to believe that this should be different for DBT. However, the trained radiologist can adapt when analyzing different data sets, indicating that high-performance deep learning models still lack the “key” characteristics that differentiate the disease from normal [107].

Image analysis performance is enhanced by the use of the following architectures: AlexNet, VGGNet and ResNet, YOLO or U-net that we describe below:

AlexNet was proposed by Krizhevsky et al. [97] for the ImageNet Large Scale Visual Recognition Challenge (ILSVRC) in 2012 [39].

AlexNet [103] consists of 8 layers, 5 layers of convolution and 3 dense, fully connected layers, overlapping overlay, abandonment, data augmentation, ReLU activations after each convolutive layer and fully connected, SGD with impulse [97]. AlexNet is used for image recognition in image analysis and is usually applied to issues involving semantic segmentation and high-resolution data classification tasks [39,70,73].

VGG (Visual Geometry Group): Consists of 13 convolution layers (in VGG16) & 16 convolution layers (in VGG19), 3 dense layers, pooling and three RELU units, very small responsive fields [108]. VGG is used for object recognition, classification of medical images [109,110] and image segmentation [18,36] VGG loses accuracy when the depth becomes too high.

ResNet (Residual Neural Network): Contains closed units or closed recurring units and has a strong similarity to recent successful elements applied in RNNs [103]. ResNet is characterized by: residual mapping, identity function, and a two-layer residual block, one layer learns from the residue, the other layer learns from the same function and has high level of performance in image classification [111] and audio analysis tasks [39,112].

GoogLeNet is built from 22 deep LAYERS CNN and 4 million parameters and contains several layer filters and stacked convolution layers [113]. It was used for batch normalization, image distortions, and RMSprop [103].

U-Net, developed by Ronneberger [101], addresses the problem of locating images of a standard CNN by extracting data features followed by reconstruction of the original dimension through an upsampling operation. U-Net is a type of Enconder-Decoder network in which the codification output belongs to the input space. U-Net is used in single-stage segmentation and classification [114], specifically in the location of cancerous lesions [38,115,116]. SegNet [39,117] is a U-Net variant that uses maximum grouping indices in the upsampling step that reduces the complexity of U-Net space [118].

RNNs were developed by Rumelhart et al. [119] using with efficiency the correlations existing between input data of a prediction problem, through which they process sequential data in relation to text analysis [84,119,120] in electronic medical records to predict diseases [121,122] and speech recognition [123]. RnN variants are: one-way, learning from the past and predicting the future and bidirectional that uses the future to restore the past. RNN has the following variants: LSTM, GRU, Recursive NNs and two-way RNNs (BiRNN). LSTMs were introduced by Hochreiter and Schmidhuber [39,103,124] and consist of: the gate of oblivion that alleviates the escape and explosion gradient, the entrance gate and the exit gate, the last two track the flow of data coming in and out of the cell. They were used in speech recognition [45], path prediction [46] and medical diagnosis [64], in which the authors proposed an LSTM network, called DeepCare, combining different types of data to identify clinical diseases.

GURs (recurrent unit gated) created by Kyunghyun Cho et al. in 2014 [48], solve the problem of increasing the time complexity of LSTM, when large amounts of data are used. The GRU consists of a reset gate in which it is decided how much information from the past is transmitted in the future, and an update gate that decides how much information from the past can be forgotten. GRU and LSTMs have similar applications especially in speech recognition [39,125].

The two-way recurring neural network and the Boltzmann BRNNs introduced by Schuster and Paliwal [44] are characterized by the fact that the hidden state is updated by using past information, as in a classic RNN, and by using information related to future moments. They were applied in handwriting and speech recognition, where they are used to detect missing parts of a sentence in a knowledge of the other words [41,126]. BM models are a family of RNNs that are easy to implement and that reproduce many probability distributions, BMs are used in image classification. BMs combined with other models are used to locate objects [39,40,127]. In the classification of images, BMs are used to identify the presence of a tumor [128]. BM models are slow and ineffective when the data size increases exponentially due to the complete connection between neurons [129]. A restricted BM was proposed in which relaxing the connections between neurons of the same or one-way connection between neurons would solve the problem of the classic BM model [5].

AEs, developed by Rumelhart et al. [119], consisting of encoder and decoder, with the aim of reducing the size of the data through significant representations and learning data characteristics for the reconstruction of outputs. They are used in applications in medical image analysis [72,130], natural language processing [67] and video analysis [68].

Additional variants of AE that can be found in the literature are variational AE (VAE). In a VAE, the encoder is represented by the probability density function of the input into the feature space and, after the encoding stage, a sampling of the new data using the PDF is added. Differently from the DAE and the SAE, a VAE is not a regularized AE, but is part of the generation class [39].

GAN it is used to generate synthetic training data from original data using latent distribution [131]. It consisted of two networks, a generator estimates false data from input data, and a discriminator, which differentiates fake data from real data and separates it in order to increase the quality of the data generated. GAN has two problems: the problem of the collapse of the mode, and the fact that, can become very unstable [103].

DBN: The DBN (Deep Network of Beliefs), created by Hinton [132], consists of two networks that build each other: of beliefs represented by an acyclic graph composed of layers of stochastic binary units with weighted and respectively weighted connections, restricted Boltzmann Machines which is a stochastic. DBNs are applied in image recognition and speech recognition, in classification to detect lesions in medical diagnosis and, in video recognition to identify the presence of persons [133], in speech recognition to understand missing words in a sentence [134] and in application on physiological signals to recognize human emotion [39,135,136].

DTN contains a characteristic extraction layer, which teaches a shared feature subspace in which marginal source distributions and target samples are drawn close and a layer of discrimination that match conditional distributions by classified transduction [103,106].

TDSN contains two parallel hidden representations that are combined using a bilinear mapping [137]. This arrangement provides better generalization compared to the architecture of a single module. The prejudices of the generalizers with regard to the learning set shall be inferred. It works effectively and better than an eco-validation strategy when used with multiple generalizers compared to individual generalizers.

DIM maximizes mutual information between an input and output of a highly flexible convolutive encoder [103,138] by forming another neural network that maximizes a lower limit on a divergence between the marginal product of encoder input and output. Estimates obtained by another network can be used to maximize the reciprocal information of the features in the input encoder. The memory requirement of the DIM is lower because it requires only encoder not decoder.

### 4.2. Combinations of Different DL Models Depending on the Type of Data Involved in the Problem to Be Solved

DL models can be combined in five different ways depending on the type of data involved in the problem to be solved. Of these, three types of HA (hybrid architectures), namely the integrated model, the built-in model and the whole model.

In the integrated model, the output of the convolution layer is transmitted directly as input to other architectures to the residual attention network, the recurrent convolutive neural network (RCNN) and the model of the recurrent residual convolutive neural network (IRRCNN) [103,139].

In the built-in model (the improved common hybrid CNN-BiLSTM), the size reduction model and the classification model perform together, the results of one represent the inputs for the other model. In the model (EJH-CNN-BiLTM), several basic models are combined.

In the transfer learning model (TL) is trained and uses the same type of problem. CNN models that use the TL model are VGG (e.g., VGG16 or VGG19), GoogLeNet (e.g., InceptionV3), Inception Network (Inception-v4), Repiuled Neural Network (e.g., ResNet50), AlexNet. Joint AB based DL combines max pooling, and careful sharing [103].

### 4.3. Combinations of Different DL Models to Benefit from the Characteristics of Each Model with Medical Applications Are: CNN + RNN, AE + CNN and GAN + CNN

CNN + RNN are used for the capabilities of the CNN feature extraction model and the RNNs [140]. Because the result of a CNN is a 3D value and an RNN works with 2D-data, a remodeling layer is, associated between CNN and RNN, to convert production of CNN into an array. CNN + RNN have been successfully applied in text analysis to identify missing words [141] and image analysis to increase the speed of magnetic resonance image storage [49,50]. CNN + RNN variants are obtained by replacing the Standard RNN component with an LSTM component [39,48,65].

AE + CNN architecture combines AE as a pre-training model when using data with high noise levels, and a CNN as a feature extractor model. AE + NVs have an application in image analysis to classify noisy medical images [76] and in the reconstruction of medical images [86,130].

GAN + CNN combines GAN as a pre-workout model to moderate the problem of over-mounting, and a CNN, used as a feature extractor. It has applications in image analysis [39,88,142].

The DL architectures applied especially in image analysis are CNN, AE and GAN. NVs preserve the spatial structure of the data, and are used as feature extractors (especially U-Net), AEs reduce the characteristics of complex images in the analysis process, and GANs are pre-training architectures that select input categories to control overfitting.

U-Net + Kite-Net + Attention U-Net + HarDNet-MSEG ahitecture, the DL model imagined by Luca, A.R. & all [143], combined model it designed takes into account the key features of the architectures involved: U-Net will be enhanced with a block context aggregation encoder and still retains the low-level image features that result from U-Net, but will generate slightly finer segmentation without adding costs due to context aggregation blocks; Kite-Net will contain a unit with attention gates and a Kite-Net decoder, in this way add a benefit of attention to the details of Kite-Net; a partial decoder like the one in the HarDNet-MSEG architecture used as the new U-Net decoder to reduce training time; U-Net Attention that suppresses irrelevant regions, key features, does not add significant computing costs, with a slightly smoother segmentation of image features. This combined DL model is not demonstrated in practice being a project [143,144]. 

### 4.4. Applications in Medicine and the Performance of DL Models Depending on the Therapeutic Areas in Which They Were Used

We further highlight the acquisitions in the study of deep learning and its applications in the analysis of the medical image [41]. You can easily identify references to image labeling and annotation, developing new deep learning models with increased performance, and new approaches to medical image processing:

- diagnosis of cancer by using CNN with different number of layers [145],
- studying deep learning optimization methods and applying in the analysis of medical images [146],
- development of techniques used for endoscopic navigation [147],
- highlighting the importance of data labelling and annotation and knowledge of model performance [148,149],
- perfecting the layer-wise architecture of convolution networks [103], lesson the cost and calculation time for processor training [150],
- description of the use of AI and its applications in the analysis [103] of medical images [151],
- diagnosis in degenerative disorder using deep learning techniques [152] and,
- detection of cancer by processing medical images using the medium change filter technique [153],
- classification of cancer using histopathological images and highlighting the rapidity of Theano, superior tensor flow [153],
- development of two-channel computational algorithms using DL (segmentation, extraction of characteristics, selection of characteristics and classification and classification, extraction of high-level captures respectively) [154],
- malaria detection using a deep neural network (MM-ResNet) [155].

We will exemplify in Table 2 [37] applications in medicine and the performance of DL models depending on types of medical images and the therapeutic areas in which they were used.

## 5. Description of Methods for Incorporating Data Types and the Applications in Which They Are Used

### 5.1. Schematically Present the Methods of Knowledge Incorporation and the Types of Data Used for DL Objectives in the Interpretation of Medical Images

**Figure 5:**
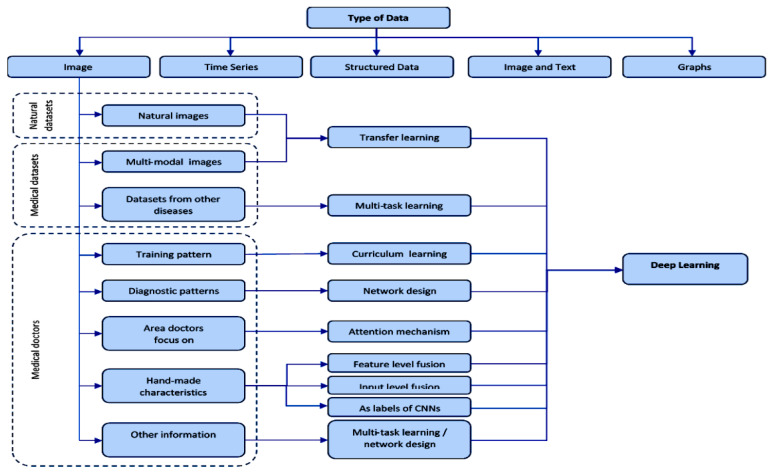
Knowledge incorporation methods and data types used for DL objectives in the interpretation of medical images.

We will exemplify the methods of incorporation of medical knowledge and data according to the purpose of DL models in medical applications, namely, diagnosis-classification, detection, segmentation, reconstruction and recovery of medical images, generation of medical reports, see Figure 5.

### 5.2. Classification in Medical Images

#### 5.2.1. Methods of Incorporating Information

Transfer learning uses multimodal medical images and natural images.

Multitask learning uses medical data from other diseases.

Curriculum learning uses pattern training to incorporate medical data from doctors.

Network design uses diagnostic pattern from medical data from doctors.

Attention mechanism used areas doctors focus on from medical data from doctors.

Decision level fusion uses features doctors focus on from medical data from doctors.

Multi-task learning/network design used from medical data from doctors 

#### 5.2.2. Methods of Incorporation of Medical Data from Doctors for Diagnosis and Classification

Imaging doctors when interpreting medical images use patterns or procedures in diagnosing diseases. Incorporating these patterns and procedures from physicians into deep learning networks increases their performance.

Types of medical data used in deep learning models for diagnosing the disease:

- paternal training,
- paternal diagnosis,
- target regions,
- hand crafted features (appearance, structures, shapes),
- related diagnostic information
- other types of diagnostic-related information

(1). The training model consists in the curricular learning through which tasks, images evolve from simple to complex in the training process. The curriculum involves a suite of training samples classified in ascending order of learning difficulty. The training model through curricular learning introduced into the deep learning network is developed by [162].

(2). General models of diagnosis of doctors, namely, the patterns and procedures used by imaging doctors when interpreting medical images. Radiologists diagnose imaging in three stages in the interpretation of X-ray images of the chest: overview, local lesion regions and subsequently combine general data [163].

(3). The use of the diagnostic pattern of radiologists for the diagnosis of thoracic disease) by extracting and combining global and local traits is carried out in [163]. Target regions or “attention maps”. Imaging doctors focus on specific areas in the diagnosis of diseases, “warning maps”, which indicates the target areas when interpreting images.

(4). Attention features (appearance, structure, shapes), “handcrafted characteristics”, as they are made by doctors, can be described characteristics, asymmetry, edge, color, margin, shape, micro-calcification and echo pattern, acoustic attenuation, side acoustic shade, and also benign-malignant risk of pulmonary nodules is classified by six characteristics of nodules: calcification, sphericality, edge, spiculation and texture and other.

(5). Related Diagnostic Information (Merger at Decision Level, Characteristics Level Fusion, Imput-Level Fusion, Features as Labels).

Merger at decision-level. The CNN classifier model automatically extracts and combines by merger at the decision-making level of handcrafted characteristics and extracted characteristics (contrast, texture, spiculation of the image) from CNN, by merger-level decision-level results from two classifiers [164].

Characteristic-level fusion. Feature-level fusion model combines two handcrafted features, parameter less threshold adhesion statistics and gray-level co-occurrence matrix, with the five groups of deep learning features extracted from five different deep models [18,37].

Input-level fusion. Input-level fusion is achieved by the fact that handmade features are used as patches that describe specific features and are used as input for CNN followed by combination in solving the problem. In some models these patches are used as input into DScGAN to increase diagnostic performance.

Using features as labels of CNN. Image classification labels and labels of handmade features are included into deep learning patterns through the multi-task learning arhitecture to increase their performance.

(6). Other Types of Diagnostic-Related Information (Additional Labels, Additional Clinical Diagnostic Reports).

These are represented by additional labels and clinical diagnostic reports. Type of additional category labels for medical images, normal, malignant or benign, condition of the lesions is incorporated into a multi-task learning structure can improve the performance of the diagnosis of major classification load [18].

Additional clinical diagnostic reports. The clinical report is a summary of descriptions of the doctor made during the imaging examination. 

### 5.3. Detection in Medical Images

We can exemplify four categories:

- paternal training,
- paternal diagnosis,
- target regions,
- hand crafted features (appearance, structures, shapes).

#### 5.3.1. Paternal Training Is the Resolution of Tasks with Increasing Difficulties That Use Curricular Learning to Identify and Locate Lesions in Medical Images

CASED performs adaptive curriculum sampling to solve the problem of highly data imbalance and makes it possible for the model to distinguish nodules from immediate proximity and subsequently enlarges the hard-declassified global context, up to uniform categories in the empirical data pool. In this way, CASED is the most performant and is used in the detection of pulmonary nodules in thoracic CT [165].

LUNA16 also based on curricular learning is used in the detection of cardiac [166].

#### 5.3.2. Paternal Diagnosis

Radiologists use patterns to locate lesions in medical images, namely:

1. Combine images in different settings (brightness and contrast),
2. Uses bilateral, transverse, adjacent images,
3. Radiologists combine collected images in different settings (brightness and contrast) to locate lesions by visual interpretation of CT images. In the same way is built a model with multi-viewing features (FPN) brightness and contrast, combined later using an attention module that identifies the position with an increase in accuracy compared to NIH DeepLesion [167].
4. Bilateral information is compared by radiologists when interpreting images.

#### 5.3.3. Handmade Characteristics 

Handmade characteristics, e.g., locations, structures, shapes are represented by “Hand-Crafted Characteristics” for Identifying target objects, nodules or lesions in medical images.

#### 5.3.4. Target Regions

The description of the target regions, e.g., information, radiological reports, additional labels is extracted from the radiological information and coupled with the curricular learning and the results are used by the network in the ascending order of the difficulties.

### 5.4. Segmentation of Lesions and Organs into Medical Images

#### 5.4.1. Incorporation of Data from Natural Datasets or Medical Data Sets

Transfer learning uses data from natural images for performance in the segmentation of the medical image. The transfer of the acquired data of a CNN arhitectures originally trained for segmenting WM hyper-intensity on old low-resolution data to new data from the same scanner, but with good image resolution is studied by [168].

Multimodal learning in which MRI, CT, are used simultaneously by pre-trained architecture deep learning.

#### 5.4.2. Incorporation of Knowledge from Doctors

Training pattern. For the segmentation of lesions into medical images deep learning models used curriculum learning.

Diagnostic pattern. Specific patterns used by doctors and embedded in the network.

Characteristics of the image (shape, location, topology).

Radiologists rely on certain characteristics of the image, shape, position, typological lesions, when interpreting medical images.

There are three types of incorporation of features injuries from medical imaging in deep learning architectures:

1. incorporating the characteristics of the lesions in the post-processing stage,
2. incorporating the characteristics of the lesions as elements of regularization in the loss function,
3. learning the characteristics of the lesion through generational models.

#### 5.4.3. Incorporation Handmade Characteristics from Doctors

For input fusion, handmade characteristics are transformed into input patches, subsequently, the original image patches and the tagged patches are inserted into a deep segmentation network [18].

### 5.5. Reconstruction of Medical Image

The objective is to reconstruct a diagnostic image from a series of measurements. 

### 5.6. Recovery of Medical Image

Deep learning architecture use knowledge from natural images (pre-trained VGG model based on ImageNet) or medical data.

### 5.7. Generating Medical Reports

The deep learning models for image subtitles have been successfully applied for the automatic generation of medical reports [169,170]. Some templates in radiologist reports are used during the sentence generation process [80,167].

Model-agnostic method attempts to learn the short description of the text to explain this decision-making process [171] and transfer the visual characteristics of medical images to a graph of anomalies [18].

Module to incorporate the pre-built graph on multiple findings of the disease to help generate reports by using the IU-RR dataset [18,172].

### 5.8. Applications in Medicine, Methods of Incorporation of Types of Data, Datasets and Their Correlation

Imaging doctors combine data from different stages and experiences as opposed to DL models that incorporate the same types and modes of handcrafted features. Data quality and volume, annotations and labels, identification and automatic extraction of specific medical terms can help deep learning models perform in the tasks of image analysis [18] Simultaneous incorporation of different medical knowledge types features, labels, into DL architectures increases their performance (see Table 3) [102].

## 6. Conclusions

In this paper, as a research novelty, we approached in a unitary way, the constituent elements of DL models:

- Updated presentation of data types, DL models used in medical image analysis;
- Correlation and contribution to the performance of DL models of the constituent elements: data type, incorporation methods and DL architectures;
- Features and “key” tasks of DL models for the successful completion of tasks in applications in the interpretation of medical images.

The quality of the data and their volume, annotations and labels, the identification and automatic extraction of specific terms, from reports, guides, books in the medical field, can increase the diagnostic accuracy of doctors and help deep learning models perform in the tasks of image analysis. Doctors use a descriptive language, namely, contour, contrast, appearance, localization, topology, etc., or compare bilateral images. The incorporation of these representations, attributes from images, in DL architectures increase their performance.

Imaging doctors combine data from different stages and experiences as opposed to DL models that incorporate the same types and modes of handcrafted features. Data quality and volume, annotations and labels, identification and automatic extraction of specific medical terms can help deep learning models perform in the tasks of image analysis [18]. Incorporating these features, labels, into DL architectures increases their performance [102].

The diagnostic model, the training model simultaneously incorporates high-level and low-level knowledge (handcrafted features, anatomical priorities). High-level medical data is incorporated as input images, and low-level medical data is learned using specific network structures [18,237] and along with direct networking, information from low-level medical data can also be used to design training commands when combined with the easy-to-use training model [18,173]. Simultaneous incorporation of different medical knowledge types can increase performance of deep learning patterns in medical applications.

DL can be a support in solving complex problems, with uncertainties of options in investigations and therapy and could help medically and by filtering, providing data from literature. This aspect leads to a personalized medicine of the patient’s disease with diagnostic and therapeutic options based on scientific evidence. Another aspect is represented by the time encoded by the doctor in patient care, time gained by the constructive and effective support of DL in medical decision-making and synthesis activities.

The use of “key” characteristics specific to each constituent of DL models and the correct determination of their correlations, may be the subject of future research, with the aim of increasing the performance of DL models in the interpretation of medical images.

## 7. Research Problems

Problems in medical image analysis can be categorized as follows:

- identification and automatic extraction and standardization of specific medical terms,
- representation of medical knowledge,
- incorporation of medical knowledge.
- Problems in medical image analysis are related to:
- medical images provided as data for deep-street models require: quality, volume, specificity, labelling.
- providing data from doctors, descriptive data, labels are ambiguous for the same medical and non-standard references.
- laborious time in data processing are problems to solve in the future.
- lack of clinical trials demonstrating the benefits of using DL medical applications in reducing morbidity and mortality and improving patient quality of life [39,102,264,265].

Full analysis of the mechanism of realization of medical applications, from data, databases, methods of incorporation of knowledge into DL models and improvement of DL models to their performance transposed into medical applications lead to the following problems to be solved: identification and automatic extraction of specific terms from medical documents, representation of medical knowledge, incorporation of medical knowledge.

Specific medical terms and descriptive attributes corresponding to diseases in medical images, by incorporating in DL models improve their performance and therefore involve solving problems related to the identification and automatic extraction of specific terms from medical documents, the presentation of medical knowledge, the incorporation of medical knowledge.

Problems in medical image analysis are related to quality, volume, specificity and data labelling in medical images used for a particular action by DL. Also, the provision of data from doctors, handmade, ambiguous expressions for the same medical references, uncertain limits of segments in images, low resolution of images, annotations, labels and laborious time in data processing are problems to solve in the future.

Another problem is the lack of clinical trials demonstrating the benefits of using DL’s medical applications in reducing morbidity and mortality and improving the quality of life of patients.

## 8. Future Challenges

These consist of domain adaptation, knowledge graph, generational models, and network architecture search techniques.

The adaptation of the domain consisted of transferring information from a source domain to a target domain, such as adversarial learning [266], makes it narrow the domain change between source and target domain in input space [267], feature space [268,269] and output space [270,271]. It can be used to transfer knowledge of one set of medical data to another [212] even when they have different modes of imaging or belong to different diseases [18,168,272]. UDA (unsupervised adaptation of the field) that uses medical labels has demonstrated performance in disease diagnosis and organ segmentation [18,81,188,255,273].

The knowledge graph, which has the specificity of incorporating multimodal medical data achieves performance in the analysis of the medical image and the creation of medical reports [167]. The graphs of medical data describing, the relationship between different types of knowledge, the relationship between different diseases, the relationship between medical datasets and a type of medical data, help the models of deep learning to perform [274].

Generative models, GAN and AE are used for segmentation tasks in particular. GAN uses MRI datasets for CT image segmentation [18,225,272]. GAN is a type of unsupervised deep learning network used in medical image analysis. AE are used in extracting characteristics, shape priorities in objects such as organs or lesions, completely unsupervised and are easily incorporated into the process of network training [18,85,237].

In traditional machine learning, the common learning process is separated and is carried out only on certain models, data sets and tasks. Therefore, knowledge is not retained or transferred to each other models. Instead, in deep learning, transfer learning can use knowledge such as the weights and characteristics of a pre-trained model to prepare a new model, as well as to address problems in the task that has a smaller amount of data. Transfer learning with deep learning patterns is faster, has improved accuracy and/or needs less training data [275].

A new approach to transfer learning, to address the problem of lack of data training in medical imaging tasks is represented by the technique of learning by transfer called dual transfer learning. Using the characteristics learned to improve the performance of other tasks by, such as classification in skin lesions, such us, benign and malignant or in the case of breast lesions to classify histological mammary images into four classes: invasive carcinoma, in situ carcinoma, benign tumor and normal tissue [276].

Using cloud computing provides a solution for managing the enormous amount of data. It also helps to increase efficiency and reduce costs. In addition, it offers the flexibility to train DL architectures [104].

With the recent development of computing tools, including a chip for neural networks and a mobile GPU, we will see more deep learning applications on mobile devices. It will be easier for users to use DL [104]. 

Network Architecture Search Technique (NAS) can automatically identify a certain network architecture in computer vision tasks [277] and promises that use and performance in the medical field [18,278].

With audacity, hope and confidence in the realization of our scientific desires we, authors, we launch an appeal to the international scientific forum with the aim that the following ideas will be put into practice at the initiative of some standard researchers in the field, “voices heard and heard” and who have the power to flesh them out:

- the establishment of a federation institution integrating scientific data and products specific to the field;
- value categorization of industry-specific achievements;
- launching challenges to be developed and completed;
- facilitating the free circulation of discoveries, methods, formulas of scientific products within this federation institution;
- establishing the board of the federation institution through the input and integration of “consequential brains” in the field;
- the creation of a Hub of Ideas under coordination within the federation board with assignment of themes for development on specific teams;
- joint effort for an idea launched within the federation institution;
- an inventory of functional applications and methods, performing in the specific field;
- the creation of a financing system to support and implement ideas specific to the field;
- integration of researchers with notable ideas and performance limited funding or access to knowledge by belonging to geographical areas or institutions under represented internationally in the specific field.

## Figures and Tables

**Table 1:** Medical applications of DL models according to the scope for which they were used [39].

Task	Contribution	Model
Classification	Benefit from unlabelled data for lung tumour stratification	DBN [40]
	Introduction of a transfer learning approach in rectal cancer prediction	CNN [41]
	Identification of bladder tumour sub-types from histopathological images	ResNet [42]
	Improvement in breast tumour estimation by considering a large set of risk factors	CNN [43]
	Estimation of the cancer grade	CNN [44]
	Estimation of the cancer type	CNN [45,46], ResNet [47]
	Limitation of overfitting	GAN [48], ResNet [49]
	Analysis of the particular characteristics of the heart by using echocardiograms	ResNet [50]
	Improvement in bone image quality	U-Net [51]
	Analysis of the impact of gender on skeletal muscles	CNN [52]
	Automatic estimation of brain diseases risk	AlexNet [53], CNN [54]
	Improvement of accuracy and efficiency in COP diseases	ResNet [55], VGGNet + CNN [56], DBN [57]
	Analysis of interstitial lung diseases	CNN [58]
	Estimation of the normal levels of the pancreas	CNN [59,60]
	Improvement in image quality	CNN [61], CNN + LSTM [62]
	Improvement in accuracy in abdominal ultrasounds	CNN [63]
Detection	Optimal localization of lung cancer sub-types	CNN [64]
	Low-cost object detection for malaria	YOLO [65]
	Improvement in image accuracy in neoplasia analysis	ResNet [66]
Segmentation	Analysis of colour contrast and parameter variability issues in pancreatic tumour	U-Net [67]
	Impact of dimension variations on DL model performance in thyroid melanomas	U-Net [68]
	Limitation of the overfitting problem in bone cancer	CNN [69], GAN + U-Net [70]
	Improvement in image accuracy in lung and prostate cancer	U-Net [71,72], GAN [73]
	DL model for multi-step integration and registration error reduction in atrial fibrillation analysis	CNN + LSTM [74]
	Accuracy in the analysis of irregular pelvic hematoma images	U-Net [75]
	Improvement in aortic disease analysis with the introduction of new accuracy measures	U-Net [76]
	Introduction of the transfer learning approach in atrium study	U-Net [49]
	Analysis of the impact of the image quality in osteoarthritis	U-Net [77], RCNN [78]
	Introduction of transfer learning and attention mechanism in the study of the knees	VGGNet + U-Net [79]
	Improvement in image accuracy of the cartilage	U-Net [80], HNN [15], U-Net + GAN [81], RCNN
	Combination of the region-based approach with U-Net for bone diseases	RCC + U-Net [82]
	Limitation of overfitting in White Matter analysis	GAN [83]
	Colour quality improvement in orbital analysis	U-Net [84]
	Segmentation of lung lobe using different types of datasets	U-Net [85]
	Analysis of image effects in neoplasia and catheter detection	U-Net [66], RNN [86]
Reconstruction	Improvement in the Signal-to-Noise Ratio Multi-data integration	CNN [87]
	Improvement in image quality at high levels in the study of coronary diseases	CNN [88]
	Application of CNNs to computed tomography for chest digital images	CNN [89]
	Introduction of a DAE as a priori model for noise density in magnetic resonance	DAE [90]
	Analysis of perturbation effects	CNN [91]
	Introduction of transfer learning into magnetic resonance	CNN [92]
	Limitation of overfitting	CNN + GAN [93]

Acronyms: Deep Network of Beliefs (DBN), Generative Adversarial Network (GAN), Tensor Deep Stacking Network (TDSN), Convolutional Neural Network (CNN), Visual Geometry Group Network (VGG Net), Fully Convolutional Network (U-Net), Residual Neural Network (ResNet), You Only Look Once (YOLO), Recurrent Neuronal Network (RNN), Long Short-Term Memory (LSTM).

**Table 2:** Objectives and performance of DL models in medical applications classified according to the therapeutic areas.

	Type of Data	Sample	Objective	Model Design	Results	Therapeutic Area	Paper
**Mammography**	Mammography images	45,000 images	Diagnosis of breast cancer	CNN	AUC of 0.90	Oncology	[40]
	Mammography	667 benign, 333 malignant	Diagnosis of early breast cancer	Stacked AE	AUC of 0.89	Oncology	[127]
	Mammography images, biopsy result of the lesions	600 images biopsy	Differentiation benign lesions one malignant masses	CNN	AUC of 0.80	Oncology	[125]
	Mammography images	840 mammograms images	Evaluate the risk of coronary disease used breast arterial calcification classifier	CNN	Misclassified cases of 6%	Cardiovascular	[71]
	Digital mammograms	661 digital images	Estimation of breast percentage density	CNN	AUC of 0.981	Oncology	[80]
	Mammography images	Mammograms from 604 women	Segment areas in the breast	CNN	AUC of 0.66	Oncology	[49]
	Digital mammograms images	29,107 mammograms images	Probability of cancer	CNN	AUC of 0.90	Oncology	[87]
**Ultrasound**	Image of the heart 2D	400 images with five different heart diseases and 80 normal echocardiogram images	Segment left ventricle images with greater precision	Deep belief networks	Hammoude distance of 0.80	Cardiovascular	[77]
	Ultrasound images	306 malignant tumor images, 136 benign tumors images	Detect and differentiate breast lesions with ultrasound	CNN, AlexNet, U-Net, LeNet	0.91 and 0.89 depending on the data	Oncology	[65]
	Transesophageal ultrasound volume and 3D geometry of the aortic valve images	3795 volumes from the aortic valves from 150 patients	Diagnose, stratification and treatment planning for patients with aortic valve pathologies	Marginal space deep learning	Position error of 1.66 mms and mean corner distance error of 3.29 mms	Cardiovascular	[45]
**Radiography**	Radiography images	7821 subjects	CAD for diagnosis of knee osteoarthritis	Deep Siamese	AUC of 0.66	Traumatology	[141]
	Radiography images	420 radiography images	Osteoarthritis diagnosis	CNN	AUC of 0.92	Traumatology	[81]
	Radiographs	112,120 frontal view chest17,202 frontal view chest radiographs with abinary class label for normal vs abnormal	Abnormality detection in chest radiographs	CNN	AUROCs of 0.960 and 0.951. AUROCs of 0.900 and 0.893	Radiology	[78]
**Slide image**	Pathology cancer images (hematoxylin and eosin)	5202 images tumorinfiltrating lymphocytes	Study of tumor tissue samples. Localize areas of necrosis and lymphocyte infiltration	Two CNNs	AUC of 0.95	Oncology	[118]
	Giemsa-stained thin blood smear slides cell images	27,558 cell images	Screening system for Malaria	CNN	AUC of 0.94	Infectious Disease	[121]
	Microscopy image patches	249 histologic images	Classification of breast cancer histology microscopy images	CNN and SVM	AUC of 0.77–0.83 for carcinoma/noncarcinoma classification	Oncology	[134]
	Microscopy histopathological images	7909 images of breast cancers	CAD for breast cancer histopathological diagnosis	CNN	AUC of 0.93	Oncology	[135]
	Microscope images	200 female subjects aged from 22 to 64	Cervix cancer screening	Multiscale CNN	Mean and standard deviation of 0.95 and 0.18	Oncology	[88]
	Whole-slide prostate histopathology images	2663 images of prostate histopathology images	Whole-slide histopathology images to outline the malignant regions	CNN	Dice coefficient of 0.72	Oncology	[78]
**Ocular fundus**	2D images	243 retina images	Diagnose retinal lesions	CNN	Precision recall curve of 0.86 in microaneurysms and 0.64 in exudates	Ophthalmology	[120]
	2D images	85,000 images	Diabetic retinopathy detection and stage classification	Bayesian CNN	AUC value of 0.99	Ophthalmology	[42]
	Images	6679 images from Kaggle’s Diabetic Retinopathy Detection	Detect retinal hemorrhages	CNN	AUC of 0.894 and 0.972	Ophthalmology	[47]
	Images	168 images with glaucoma and 428 control	Detect and evaluate glaucoma	CNN: ResNet and U-Net	AUC of 0.91 and 0.84 respectively	Ophthalmology	[128]
	Images	90,000 images with their diagnoses	Predict the evolution of diabetic retinopathy	CNN	AUC of 0.95	Ophthalmology	[51]
	Images	7000 colour fundus images	Image quality of diabetic retinopathy	CNN	Accuracy of 100 %	Ophthalmology	[52]
	AREDS (age related eye disease study) image	130,000 fundus images	Diagnosis of Age-related Macular Degeneration	CNN	94.97 sensitivity and 98.32 % specificity	Ophthalmology	[156]
	Fundus images	219,302 from normal participants without hypertension, diabetes mellitus (DM), and any smoking history	Predict age and sex from retinal fundus images	CNN	AUC 0.96	Ophthalmology	[157]
**Dermoscopy**	Images	350 images of melanomas and 374 benign nevi	Acral lentiginous melanoma diagnosis	CNN	AUC of over 0.80	Oncology	[129]
	Clinical images	49,567 images	Recognize nails nychomycosis lesions	Region-based-CNN	AUC of 0.98, AUC of 0.95, AUC of 0.93, AUC of 0.82	Dermatology	[130]
	Myocardial perfusion images	1638 patients	Obstructive coronary disease prediction	CNN	Sensitivity value of 0.82 and 0.69 for both use cases	Cardiovascular	[91]
**Arterial labeling**	Arterial spin labeling (ASL) perfusion images	140 subjects	Monitoring cerebral arterial perfusion	CNN	AUC of 0.94	Cardiovascular	[44]
**Frames from endoscopy**	Frames from endoscopy videos	205 normal and 360 abnormal images	Detection and localization of gastrointestinal anomalies	CNN	AUC of over 0.80	Gastroenterology	[72]
**Tracking dataset multi-instrument Endo-Visceral Surgery and multi-instrument in vivo**	Single-instrument Retinal Microsurgery Instrument Tracking dataset, More-instrument Endo-Visceral surgery and multi-instrument in vivo images	940 frames of the training data (4479 frames) and 910 frames for the test data (4495 frames)	Detect the two-dimensional position of different medical instruments in endoscopy and microscopy surge	Convolutional Detection regression network	AUC of 0.94	Robotic Surgery	[76]
**CT/PET-CT/SPECT**	Nuclear MRIs 3D	124 double echography	Diagnose possible soft tissue injuries	Deep Resolve, a 3D-CNN model	MSE of 0.008	Traumatology	[53]
	Retinal 3D images obtained by Optical Coherence Tomography	269 patients with AMD, 115 control patients	Retina age-related macular degeneration diagnostic	CNN	AUC of 0	Ophthalmology	[158]
	123I-fluoropropyl carbomethoxyiodophenyl nortropane single-photon emission computed tomography (FP-CIT SPECT) 2D images	431 patient cases	Automatic interpretation system in Parkinson’s disease	CNN	AUC of 0.96	Neurology- Psychiatry	[84]
	Abdominal CT 3D images	231 abdominal CT	Classify tomography and evaluate the malignity degree in gastro-intestinal stromal tumors (GISTs)	Hybrid system between convolutional networks and radiomics	AUC of 0.882	Oncology	[83]
	CT image patches 2D	14,696 images	Diagnose interstitial lung disease	CNN	AUC of 0.85	Pneumology	[46]
	3D MRI and PET	93 Alzheimer Disease, 204 MCI Mild Cognitive Impairment converters and normal control subjects	Diagnose early Alzheimer disease stages	Multimodal DBM	AUC of 0.75–0.95	Neurology- Psychiatry	[41]
**MRI**	Diffusion-weighted imaging maps using MRI	222 patients. 187 treated with rtPA (recombinant tissue-type plasminogen activator)	Decide Acute Ischemic Stroke patients’ treatment through volume lesions prediction	CNN	AUC of 0.88	Neurology- Psychiatry	[122]
	Magnetic resonance images	474 patients with schizophrenia and 607 healthy subjects	Schizophrenia detection	Deep discriminant autoencoder network	Accuracy over 0.8	Neurology- Psychiatry	[124]
	Gadoxetic acid–enhanced 2D MRI	144,180 images from 634 patients	Staging liver fibrosis through MR	CNN	AUC values of 0.84, 0.84, and 0.85 for each stage	Gastroenterology	[64]
	Resting state functional magnetic resonance imaging (rs-fMRI), T1 structural cerebral images and phenotypic information	505 individuals with autism and 520 matched typical controls	Identify different autism spectrum disorders	Denoising AE	Accuracy of 0.70	Neurology- Psychiatry	[126]
	3D MRI and PET	93 Alzheimer Disease, 204 MCI Mild Cognitive Impairment converters and normal control subjects	CAD for early Alzheimer disease stages	Multimodal DBM	Accuracy of 0.95, 0.85 and 0.75 for the three use cases	Neurology- Psychiatry	[41]
**CT/PET-CT/SPECT**	CT images, MRI images and PET images	6776 images	Classify medical diagnostic images according to the modality they were produced and classify illustrations according to their production attributes	CNN and a synergic signal system	AUC of 0.86	Various	[159]
	CT image 2D	63,890 patients with cancer and 171,345 healthy	Discriminate lung cancer lesions in adenocarcinoma, squamous and small cell carcinoma	CNN	Log-Loss error of 0.66 with a sensitivity of 0.87	Oncology	[160]
	CT 2D images	3059 images from several parts of human body	Speed up CT images collection and rebuild the data	Dense-Net and CNN	RMSE of 0.00048	Various	[142]
	CT images 3D	6960 lung nodule regions, 3480 of which were positive samples and rest were negative samples (nonnodule)	Diagnose lung cancer in low-dosage CT	Eye-tracking sparse attentional model and CNN	Accuracy of 0.97	Oncology	[90]
	CT images 2D and text (reports)	9000 training and 1000 testing images	Processing text from CT reports in order to classify their respective images	CNN	AUC of 0,58, 0,70–0.95	Various	[92]
	Computed tomography (CT)	Three datasets: 224,316, 112,120 and 15,783	Binary classification of posteroanterior chest x-ray	CNN	92% accuracy	Radiology	[161]
**MRI**	Clinical characteristics and MRI 3D	135 patients with short-, medium- and long-term survival	Predict the survival of patients with amyotrophic lateral sclerosis	CNN	Accuracy of 0.84	Neurology- Psychiatry	[67]
	Optical coherence tomography images	52,690 AMD patients’ images and 48,312 control	Differentiate Age-Related Macular Degeneration lesions in optical coherence tomography	Modification of VGG16 CNN	AUC of 0.92, AUC of 0.93 and AUC of 0.97 for the different use cases	Ophthalmology	[68]
	Lung computed axial tomography 2D images and breast ultrasound lesions	520 breast sonograms from 520 patients (275 benign and 245 malignant lesions) and lung CT image data from 1010 patients (700 malignant and 700 benign nodules)	CAD system to classify breast ultrasound lesions and lung CT nodules	Stacked denoising AE	AUC of 0.94	Oncology	[58]
	MRI 2D	444 images from 195 patients with prostate cancer	Prevent errors in diagnosing prostate	CNN	AUC of 0.94	Oncology	[88]
	MRI 2D	MICCAI 2009 left ventricle segmentation challenge database	Determinate limits between the endocardium and epicardium of the left ventricle	RNN with automatc segmentation techniqes	AUC of 1.0 in the best case	Cardiovascular	[132]
**MRI**	CT images, MRI images and PET images	6776 images for training and 4166 for tests	Classify medical diagnostic images according to the modality they were produced and classify illustrations according to their production attributes	CNN and a synergic signal system	AUC of 0.86	Various	[159]
	Functional MRI	68 subjects perform 7 activities, and a state of rest	Analyze cerebral cognitive functions	3D CNN, resting state networks	AUC of 0.94	Neurology- Psychiatry	[140]
	Liver MRIs	522 liver MRI cases with and without contrast for known or suspected liver cirrhosis or focal liver lesion	Screening system for undiagnosed hepatic magnetic resonance images	CNN	Reduces negative predictive value and leads to greater precision	Gastroenterology	[50]
	MRI images	1064 brain images of autism patients and healthy controls. MRI data from 110 multiple sclerosis patient	Evaluate the quality of multicenter structural brain MRI images	CNN	AUC 0.90 and 0.71	Radiology	[55]

Acronyms: AMD age-related Macular Degeneration, CAD Computer Aided Diagnosis, CNN Convolutional Neural Network, MRI Magnetic Resonance Images, PET Photon EmissionTomography, CT Computed Tomography, OCT Optical Coherence Tomography, D dimensions, AUC Area Under the Curve, MSE Mean Squared Error, RMSE Root Mean Square Error, DSC Dice Similarity Coefficient.

**Table 3:** Applications in medicine, methods of incorporation of types of data, datasets and their correlation.

Dataset Images	Methods of Incorporating Information	Application in Medicine
**Data doctors focus on**		
**Training pattern** high-level medical data, curriculum learning	Training modelImages with increasing complexity	diagnosis-classification of breast screening in DCE-RMN [61] application - the attention-based curriculum, used in CNN, derived from radiology reports [173] diagnosis of the proximal femoral fracture in X-ray images [18,174] diagnosing of disease [18,175,176]
**Diagnostic pattern,** low-level medical data, areas of images, characteristics of diseases	General models of diagnosis of doctors	thoracic disease diagnosis [163] final prediction of the disease [177] diagnosis chest X-ray [178] dermoscopic diagnosis of the lesion [63] achieves mass identification accuracy in the MommiNet network [179] diagnosis of skin lesions and classification of thoracic disease [180]
**Area of interest,** specific data identified by doctors, attention maps	“Attention maps” model of doctors	glaucoma diagnosis [167] classification of images of tomography with images of optical coherence of the retina (OCT) [43] diagnosis of diabetic retinopathy [93] diagnosis of esophageal fistula to radiotherapy [18,69] diagnosis of breast cancer [74] Detection of changes in lesions in melanoma screening [18,75]
**Dataset Images**	**Methods of Incorporating Information**	**Application in Medicine**
	**Attention characteristics**	
**Hand-made characteristics**	Characteristics level fusion + Incorporation level fusion	diagnosis-classification of lung nodules on CT images [18,56] diagnosis of mammary ultrasound images, [181]
	Incorporation level fusion	diagnosis of skin lesions [7] diagnosis-classification of mammographic tumor [82] diagnosis of lung nodules [18,54] diagnosis of breast cancer [182] diagnosis-classification of cardiac slices [89]
	Characteristics level fusion	diagnosis of pulmonary nodules [183] classification of breast cancer in histological images [146] diagnosis of glaucoma disease [184] diagnosis-classification of skin lesions [185] diagnosis-classification of lung nodules [18,186] diagnosis of brain tumors [187]
	Incorporation patch characteristics	
	MV-KBC	diagnosis-classification of lung nodules [56] diagnosis-classification of thyroid disease [188]
	DSc-GAN	diagnosis of thyroid nodules [18,189] diagnosis of breast cancer in multi-sequence MRI [190]
	As labels of CNNs	diagnosis-classification of lung nodules [191] differentiation (benign-malignant) of lung nodules in CT scans [8] diagnosis of glioma [192]
	**Other types of** **information**	
	Additional category label, BI-RADS label (malignant/benign)	predicts the sensitive, specific, balanced result merged for images of glaucoma in [193]
	Additional clinical diagnosis reports (abstract descriptions)	Tie-Net classifies common thoracic disease into chest X-rays [194] facilitates the interpretation of pathological images of bladder cancer [133]
**Dataset Images**	**Methods of Incorporating Information**	**Applications in Medicine**
**Natural Datasets Images**		
**Natural images** ImageNet 1 and COCO 2	**Transfer learning** - fixed feature extracts - initialization	diagnosis-detection of lymph node [18,195] diagnosis-detection of polyp and pulmonary embolism [196] diagnosis-detection of breast tumors [65] diagnosis-detection of colorectal polyps [197,198]
**Medical Datasets Images**		
**Medical images** PET CT, Mammography, X-ray, Retina-Net	Learning with more tasks (multi-task)	PET image applications are incorporated for the diagnosis-detection of lesions in CT images of the liver [25] diagnosis-detection of liver tumors [199] diagnosis-detection of breast masses [200] diagnosis-detection of pulmonary nodules in CT images [18,201] diagnosis-detection of retinal diseases in the bottom of the retina [202] diagnosis-detection colitis in CT images [203] intervertebral disc detection in X-ray images [18,204] diagnosis-detection architectural distortions in mammograms [18,205] diagnosis-detection breast tumors in mammograms [18,206] diagnosis-detection of pulmonary lung nodules in CT [207] diagnosis-detection of various lesions (e.g., liver damage, lung lesion, bone lesion, abdominal lesion) in CT images [18,208] diagnosis-detection of malignant lesions of the liver and reduce by 28% false positive average per case [18,25] diagnosis-detection of breast masses from digital tomosynthesis [200]
**Dataset Images**	**Methods of Incorporating Information**	**Applications in Medicine**
**Data doctors focus on**		
**Training pattern** high-level medical data, curriculum learning	Training model Images with increasing complexity	diagnosis-detection locates the lesion in chest [173] diagnosis-detection of pulmonary nodules in thoracic CT [18,165] diagnosis-detection of cardiac landmarks [166]
**Diagnostic pattern**, low-level medical data, areas of images, characteristics of diseases	General models of diagnosis of doctors	diagnosis-detection of lung lesions based on pneumonia COVID-19 [209] diagnosis-detection of dense vessels and ischemia [210] diagnosis-detection of thrombus [211] diagnosis-detection of hemorrhagic lesions [212] diagnosis-detection mammographic mass [213] diagnosis-detection pulmonary nodule in CT images [207]
**Area of interest**, specific data by doctors, “attention maps”	Models explicitly incorporates “attention maps”	diagnosis-detection of thoracic disease [173] diagnosis-detection of mammograms [18,214,215]
**Hand-crafted features**	Attention features	diagnosis-detection mammographic lesions [125] diagnosis detection of pulmonary nodules [216] diagnosis-detection of thyroid nodules, size and shape of the attribute of nodules [18,189] diagnosis-detection of lymph nodes in oncological imaging [217] diagnosis-detection of lung lesions [18,218]
**Dataset Images**	**Methods of Incorporating Information**	**Applications in Medicine**
**Natural Datasets Images**		
**Natural images** ImageNet 1, COCO 2, Data Set Sports-1M (1.1 million’s, video-sports) PASCALVOC dataset	**Transfer learning** - fixed feature extracts - initialization	diagnosis-evaluation of brain tumors [18,219] diagnosis-evaluation of breast tumors [200] diagnosis-evaluation of liver lesions [220] diagnosis-evaluation of lesions of the pancreas [221] diagnosis-segmentation of intimate-media limits [18,196] diagnosis prenatal segmentation of the ultrasound image [222] diagnosis-segmentation of the gland in histopathological images [18,117] diagnosis-segmentation of the proximal femur in 3D MRI [18,223] diagnosis-segmentation of multiple sclerosis [224]
**Medical Datasets Images**		
**Medical images** MRI data, CT angiography, 3DSeg-8 dataset	**Learning with more tasks (multi-task)**	diagnosis-segmentation of the left/right lung [18,225] diagnosis of cerebral disease into MRI [18,226,227] diagnosis-segmentation of heart vessel without annotations used annotated retinal images [228] diagnosis-segmentation of coronary artery, with high accuracy [18]
**Data doctors focus on**		
Deep learning: FCN U-Net GAN		diagnosis segmentation of brain [18,227] diagnosis-segmentation of skin lesions [229] diagnosis-segmentation of vessels [230] diagnosis-segmentation of anomalies in the retina fundus images [231]
**Dataset Images**	**Methods of Incorporating Information**	**Applications in Medicine**
**Data doctors focus on**		
**Training pattern** high-level medical data, curriculum learning	Training model Images with increasing complexity Self-paced learning (SPL) SPL + active learning	diagnosis-segmentation of CT images with multiple organs [232] diagnosis-segmentation in 3D pulmonary images [194] diagnosis-segmentation of liver tumors [18,212] diagnosis-segmentation of the left ventricle [18,233] diagnosis-segmenting of the finger bones [18,234] diagnosis-segmentation of vessels [18,235]
**Diagnostic pattern** LIDC-IDRI dataset, BraTS 2018 dataset	General models of diagnosis of doctors	diagnosis on uncertain nodules [236] diagnosis-segmentation of heart [237] diagnosis-segmentation of the liver [238] diagnosis-segmentation of raw tumors and clinical target volume [18,239]
**Area of interest** BRATS2015 dataset, (ImageNet, video datasets Used for 3D image segmentation)	The fusion at the feature level + concatenate	diagnosis-segmentation in histopathological images [57] diagnosis-segmentation of brain [18,240] diagnosis-segmentation of tumor brain-MRI images [18,241] diagnosis-segmentation of cellular nuclei [18,242]
**Specific characteristics** (shape, location, topology)	In the post-processing stage	diagnosis-segmentation identifying locations of breast tumors [18,243] diagnosis-segmentation anatomically [244] diagnosis-representation of anatomical cardiac form [245]
	In the loss function	diagnosis-segmentation of cardiac RM images [246] diagnostic-segmentation skin lesions [247] diagnosis-segmentation of kidney [18,248] diagnosis-segmentation of liver [18,249] diagnosis-segmentation of cardiac MRI [18,250] diagnosis-segmentation of cardiac MRI [237] diagnosis-segmentation of eye [85] diagnosis-segmentation of brain MRI [18,251] diagnosis-3D segmentation of the fine renal artery [18,252] diagnosis-segmentation of cervical cytoplasm’s [18,253] diagnosis-segmentation of scapula [18,254] diagnosis-segmentation of liver [18,255] diagnosis-segmentation of carotid [18,256] diagnosis-segmentation of head and neck [18,257]
**Dataset Images**	**Methods of Incorporating Information**	**Applications in Medicine**
**Data doctors focus on**		
**Series of measurements**	Reconstruction of medical image	magnetic resonance imaging reconstruction by compressed detection [258] image reconstruction with diffuse optical tomography (DOT) of limited angle breast cancer and limited sources in a strong scattering environment [17]
**Content-based image (CBIR)** External medical datasets and natural images	Recovery of medical image	brain tumor recovery [259] X-ray image Recovery [18,260] image recovery with chest X-ray [261] image recovery with X-ray thoracic pathology [18,262] features extracted from health areas can also be injected into the features extracted from the entire image for high recovery accuracy [18,263]
**Templates from the report of radiologist** Visual characteristics of medical images, IU-RR datasets, text templates	Generating Medical Reports	some templates from the reports of radiologists are used during the process of generating sentences [80,167] model-agnostic method to learn the short description of the text to explain this decision-making process [18,171] transfers the visual characteristics of medical images to a graph of anomalies, then retrieves text templates based on anomalies and their attributes for thoracic X-ray images [18,167] incorporate the pre-built graph (modeled with a CNN graph) on multiple findings of the disease to help generate reports by using the IU-RR dataset [18,172]
